# Thiopeptide Antibiotics: Retrospective and Recent Advances

**DOI:** 10.3390/md12010317

**Published:** 2014-01-17

**Authors:** Xavier Just-Baringo, Fernando Albericio, Mercedes Álvarez

**Affiliations:** 1 Institute for Research in Biomedicine, Barcelona Science Park-University of Barcelona, Baldiri Reixac 10, Barcelona 08028, Spain; E-Mails: xavier.just@irbbarcelona.org (X.J.-B.); albericio@irbbarcelona.org (F.A.); 2 CIBER-BBN, Networking Centre on Bioengineering Biomaterials and Nanomedicine, Barcelona 08028, Spain; 3 Department of Organic Chemistry, University of Barcelona, Barcelona 08028, Spain; 4 School of Chemistry and Physics, University of KwaZulu-Natal, 4000-Durban, South Africa; 5 Laboratory of Organic Chemistry, Faculty of Pharmacy, University of Barcelona, Barcelona 08028, Spain

**Keywords:** thiopeptides, antibiotics, natural products, peptides, heterocycles, thiazole, oxazole, dehydroamino acids, pyridine

## Abstract

Thiopeptides, or thiazolyl peptides, are a relatively new family of antibiotics that already counts with more than one hundred different entities. Although they are mainly isolated from soil bacteria, during the last decade, new members have been isolated from marine samples. Far from being limited to their innate antibacterial activity, thiopeptides have been found to possess a wide range of biological properties, including anticancer, antiplasmodial, immunosuppressive, *etc.* In spite of their ribosomal origin, these highly posttranslationally processed peptides have posed a fascinating synthetic challenge, prompting the development of various methodologies and strategies. Regardless of their limited solubility, intensive investigations are bringing thiopeptide derivatives closer to the clinic, where they are likely to show their veritable therapeutic potential.

## 1. Introduction

Since the discovery of the first antibiotics and their golden age in mid 20th century, there has been a dramatic change in the way we face the development of new antimicrobials [1,2]. At first, it seemed that the many classes of naturally occurring antibiotics could be sufficient to fight against bacterial infections, whereas, for the last few decades it was thought that semi-synthetic modifications of those natural products would be enough to overcome pathogen resistance. However, we are now facing a new age, where the discovery of novel scaffolds and new modes of action is required to fight against the emergence of resistances and cross-resistances that make previously treatable infections a new threat.

**Figure 1:**
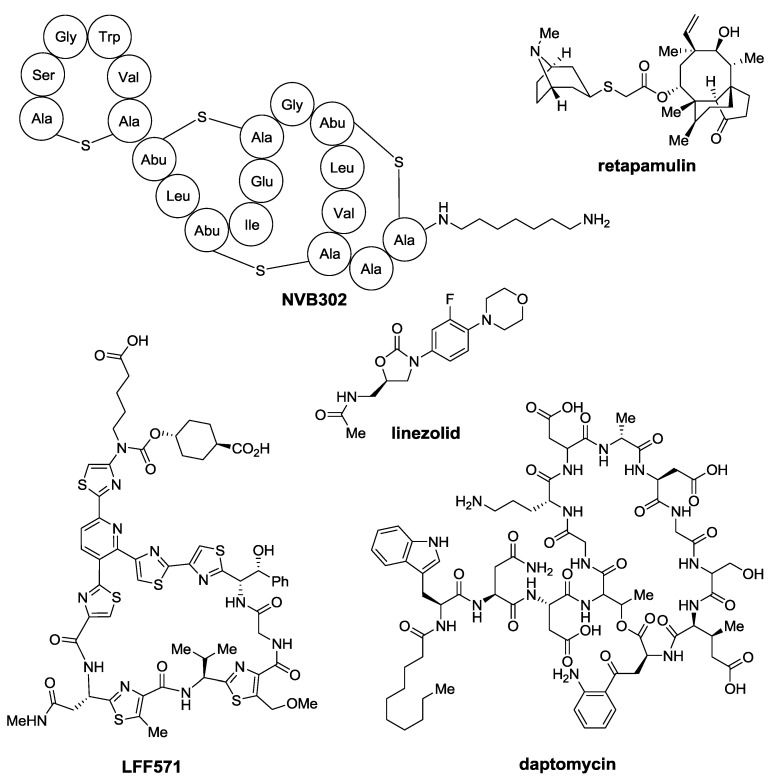
Members of new classes of antibiotics. Abu = aminobutyric acid.

Most of the antibacterial scaffolds known to date were discovered from late 1930s to early 1960s. After that period, almost forty years followed without new bactericide architectures appearing in the market. During those years, semi-synthetic modifications of the already known compounds were used to fight antibacterial resistance. However, with the new century, a batch of new antibiotic scaffolds got closer to the clinic (Figure 1). These include oxazolidinones (linezolid, 2000), lipopeptides (daptomycin, 2003) and mutilins (retapamulin, 2007). In parallel, other types of antibiotics, such as lantibiotics [3] (NVB302) and thiopeptides [4] (LFF571) [5], are under study and some members of these groups are already in clinical trials for the treatment of human infections.

Among these new families of antibiotics, thiopeptides have gathered much attention due to their potent *in vitro* activity against Gram-positive bacteria and their intriguing structures. During the last two decades, intensive investigations on known thiopeptides, their semisynthetic modification and the discovery of new members of this class of antibiotics have been the focus of the efforts of many research groups.

## 2. Thiopeptides

Thiopeptides, or thiazolyl peptides [4], are highly modified sulfur-rich peptides of ribosomal origin. They all share a series of common motifs that differentiate them from other peptide-derived and/or azole-containing natural products. Their most characteristic feature is the central nitrogen-containing six-membered ring, which can be found in many different oxidation states. This central ring serves as scaffold to at least one macrocycle and a tail, and both can be decorated with various dehydroamino acids and azoles, such as thiazoles, oxazoles, and thiazolines. All these moieties are formed though dehydration/dehydrosulfanylation of Ser, Thr, and Cys residues. Their impressive *in vitro* profile against Gram-positive bacteria, and their new mechanisms of action, have gathered the attention of many groups, both in academia and industry, as they pose an alternative to other antibiotics presently facing resistance by old pathogens. To date, more than one hundred members of this family of natural products have been identified; however, their very large molecular size and their poor aqueous solubility have been a major drawback to introduce them into the clinic. This has become their major limitation and has restricted their use to topic treatments, and so far only for pet skin infections (thiostrepton, Panolog).

**Figure 2:**
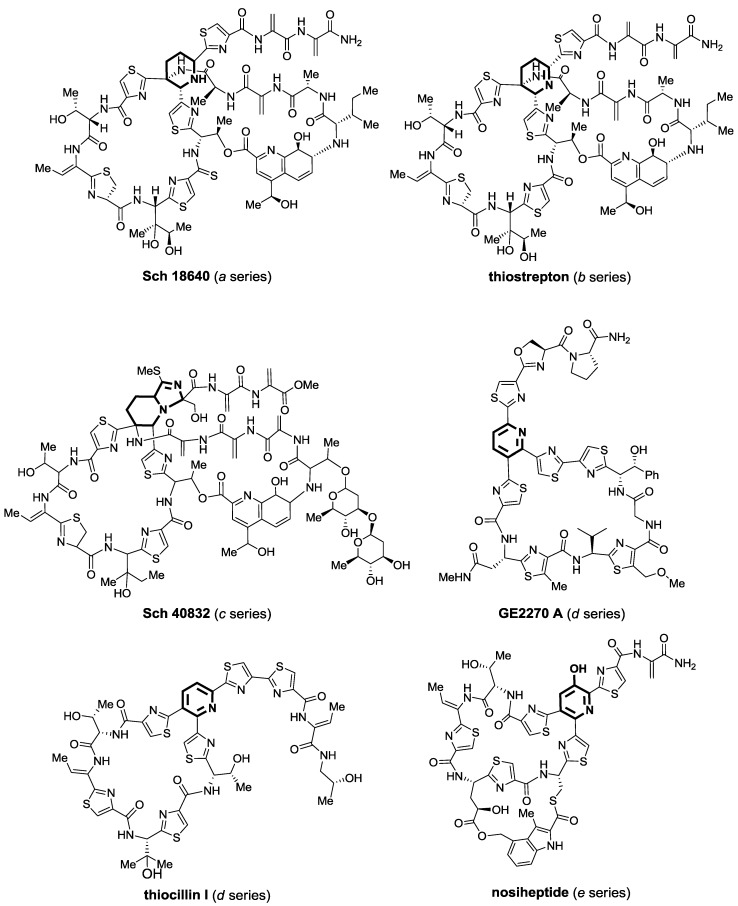
Classification of thiopeptide antibiotics into different series. Their characteristic central six-member ring is highlighted in bold.

Given the different oxidation state the central ring of thiopeptides can be found in, they have been classified into different series (Figure 2) [4]. Thus, the *a* series presents a totally reduced central piperidine, whereas the *b* series is oxidized further and contains a 1,2-dehydropiperidine ring. Only one thiopeptide of the *c* series has been isolated to date and its core moiety is somewhat unexpected, as it displays a piperidine ring fused with imidazoline. All members of series *a*, *b*, and *c* have a second macrocycle, which contains a quinaldic acid moiety. The *d* series goes further on the oxidation state rank and shows a trisubstituted pyridine ring, which is the landmark of this subgroup, the most numerous among thiopeptides. In a sense, the *e* series is even more oxidized and is easily differentiated for the hydroxyl group in the central pyridine, which is now tetrasubstituted. The *e* series also presents a very characteristic second macrocycle appending from the main one and formed by a modified 3,4-dimethylindolic acid moiety.

### 2.1. Isolation and Structure Elucidation

**Figure 3:**
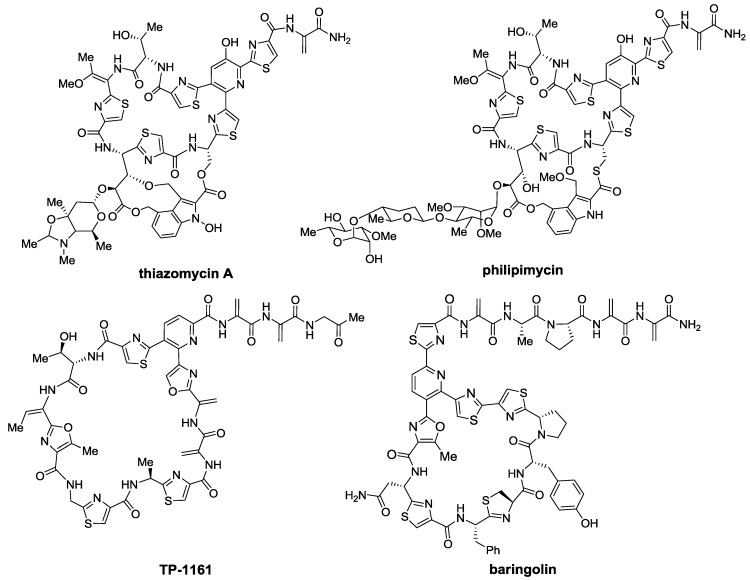
Some of the most recently described thiopeptides.

Thiopeptides have been isolated from diverse sources. In 1948, the first known member of the family, micrococcin [6], was isolated from a sample of Oxford’s sewage waters. Accounting for the highly diverse origin of thiopeptides, micrococcin P1 was more recently isolated from a completely different source, a French cheese [7]. However, more conventional samples, such as from soil are the main source of most thiopeptides. In fact, thiostrepton, the most famous member of the family, has been isolated from different soil samples [8,9,10], including one from Hawaii in 1955 [11], shortly after it was first discovered in 1954 [8,9,10]. Although a few more thiopeptides were isolated during the following years, it was from the 1980s, especially during the 1990s, that most of the known members were discovered. Nonetheless, many novel entities have also been described during the last decade. Remarkably, the first thiopeptide antibiotics isolated from a marine source were YM-266183 and YM-266184, discovered as late as 2003, in Japan [12]. During the last few years some more thiopeptides have been isolated and characterized; these include the thiazomycins (2007) [13,14,15,16], philipimycin (2008) [17], thiomuracins (2009) [18], TP-1161 (2010) [19,20], baringolin (2012) [21], and kocurin (2013) [22] (Figure 3).

The assignment of thiopeptide structures can be a very complex task, as exemplified by thiostrepton, of which structure elucidation was originally addressed by degradation studies and structure determination of fragments [23]. However, the later use of X-ray diffraction was essential to elucidate both connectivity and stereochemistry [24,25]. Although the development of NMR spectroscopy techniques has permitted the elucidation of many structures of thiopeptides, a high degree of uncertainty remains until further evidence is provided. This was clearly the case of micrococcin P1 [26]. Early studies on its constitution by hydrolysis [27,28,29] of the natural extract permitted the identification of most moieties present in micrococcin; however, there was no clear evidence of its connectivity. Later on, NMR studies [30,31,32] and synthesis of proposed structures [33,34,35,36] of the natural compound resulted in better hypotheses for its constitution and stereochemistry, although none of the synthesized products was identical to the natural one. It was not until its total synthesis was achieved by Ciufolini in 2009, 51 years after its discovery, that micrococcin P1 structure and stereochemistry were finally confirmed [37].

The structure of most thiopeptides has been investigated by a combination of degradation, mass spectrometry and NMR studies. The impossibility to obtain crystals for the vast majority of them prompted the assignment of their structure without a clear evidence of their stereochemistry. In spite of this limitation, in many cases their configuration has been proposed by analogy with similar isolates [38,39], via amino acid analysis [40,41,42] or via isotopic labeling through feeding with labeled amino acids [43,44]. In some cases, less conventional techniques have been chosen. Such are the cases of promoinducin and thiotipin, where chiral TLC was used to determine the configuration of l-Thr from an acidic hydrolysate [45,46]. Absolute configurations have also been reported after NMR spectroscopy studies and chiral capillary electrophoresis [47].

As exemplified by micrococcin P1, a synthetic approach to the problem can serve as the ultimate confirmation for both connectivity and stereochemistry. This strategy also includes the comparison of fragments with their synthetic counterparts; such was the case of GE2270A [48]. Synthesis of thiopeptides polyheterocyclic cores has been used to confirm the structure of the corresponding degradation products, while, at the same time, it has also permitted the development of the necessary synthetic methodology [49].

### 2.2. Biosynthesis

The biosynthetic pathway of thiopeptides has been very elusive for a long time; however, recent discoveries have put light on the synthesis of these highly modified peptides. Peptide-based natural products can have two distinct origins depending on how their amino acids are condensed together to form the parent peptides. These can be either synthesized on the ribosome as product of mRNA translation or can be assembled by nonribosomal peptide synthases (NRPSs). Though most highly modified peptide-derived natural products are synthesized by NRPSs, there was no evidence of such origin for thiopeptides. Surprisingly, very recent discoveries by four different groups have demonstrated that parent pre-peptide of thiopeptide is ribosomally synthesized and, thus, is genetically encoded [19,50,51,52].

**Figure 4:**
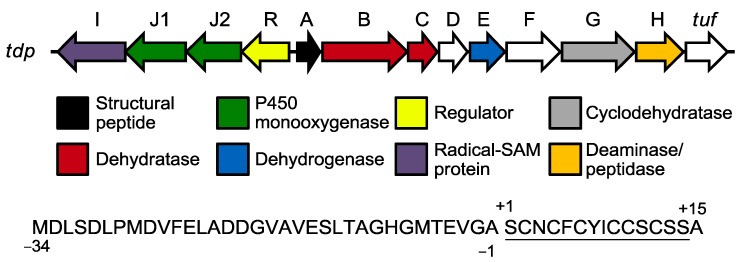
The biosynthetic gene cluster of tiomuracins and their precursor peptide sequence, which is coded in the structural gene. In the precursor peptide sequence, the structural peptide is numbered with positive figures and the leading peptide with negative ones. Residues that appear in the mature thiopeptide are underlined.

Joint efforts of bio-informatics and genome mining have been essential for the identification of genes that encode the precursor peptide and the enzymatic machinery necessary for its subsequent tailoring [18,20,50,51,52,53,54,55,56,57,58,59]. The gene encoding the precursor peptide has been identified for many thiopeptides and, in all cases, there is a perfect agreement with the expected amino acid sequence. This precursor peptide is divided in two different regions, a structural peptide of 12 to 17 residues at the *C*-terminus, which contains the amino acids that will constitute the thiopeptide itself, and a leading peptide of 34 to 55 residues at the *N*-terminus, which is cleaved during the bio-synthetic process. In some cases, the *C*-terminal structural peptide contains one or two extra residues that are cleaved during the tailoring to confer each thiopeptide its characteristic *C*-terminus [60,61]. All necessary enzymes for pre-peptide tailoring are encoded in genes surrounding that of the precursor peptide, forming a gene cluster (see Figure 4 for an example on thiomuracins’ gene cluster (*tdp*) and precursor peptide [18]; in the gene cluster, genes appear as arrows and are named with letters. Each gene (*tdpX*) codes a gene product, a protein/enzyme (TdpX)).

**Scheme 1:**
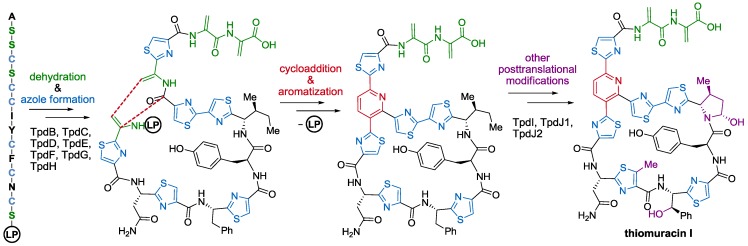
Biosynthetic pathway of thiomuracin I. LP = leading peptide. Enzymes involved in the biosynthetic pathway (TdpX) are named according to their corresponding gene (*tdpX*) in thiomuracins’ gene cluster (*tdp*).

The role of most enzymes present in some thiopeptides gene clusters has been already discovered. Similarity with known enzymes of the same function, gene deletions, and characterization of products that result from transformations with isolated enzymes, have permitted to establish which transformations and in which order they take place [51,58,59,62,63,64,65,66,67,68]. Apparently, oxazole, thiazole, and thiazoline rings are formed first through cyclization, dehydration, and, if required, oxidation of Ser, Thr, and Cys residues. In a second step, Ser and Thr phosphorylation and elimination yields the corresponding dehydroalanine (Dha) and dehydrobutyrine (Dhb) residues, respectively. Finally, intramolecular aza-Diels-Alder-like cycloaddition between distant Dha residues occurs, followed by dehydration, and, when required, elimination to constitute the central six-membered ring. Further side-chain modifications, such as oxidations, cyclizations, methylations, and incorporation of indolic or quinaldic acid moieties seem to occur in later stages of the bio-synthetic pathway (see Scheme 1 for an example on thiomuracin I biosynthesis [18]).

Both quinaldic and indolic acid moieties found in *a*–*c* and *e* series thiopeptides are synthesized from l-Trp and are part of the second macrocycle found in these compounds. This was first demonstrated by labeling [69,70,71,72] and enzyme function [73,74] experiments and, more recently, also using genetic engineering methods [63,65,66,67].

**Scheme 2:**
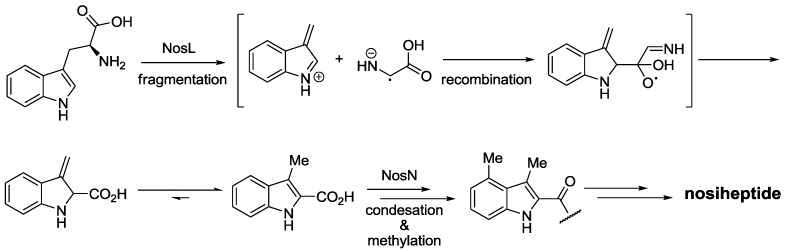
Biosynthesis of indolic acid moietiy from Trp and incorporation into nosiheptide. Enzymes involved in the biosynthetic pathway (NosX) are named according to their corresponding gene (*nosX*) in gene cluster of nosiheptide (*nos*).

In the case of indolic acid formation, Trp undergoes a radical-mediated rearrangement and Cα migrates to position 2 of indole (Scheme 2). Subsequently, *S*-adenosylmethionine-dependent 4-methylation of the aromatic scaffold after condensation with the structural peptide yields an advanced intermediate of the mature thiopeptide [65,75].

**Scheme 3:**
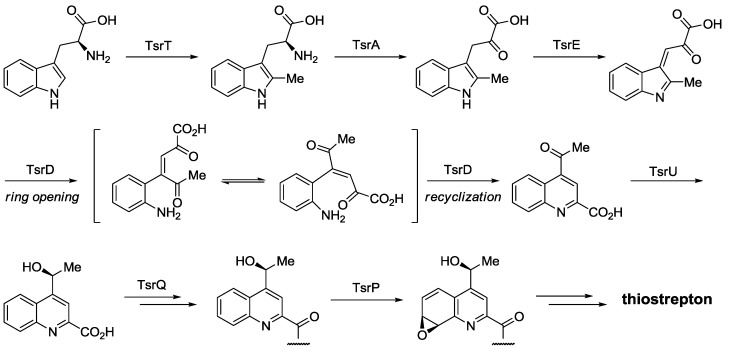
Biosynthesis of quinaldic acid moietiy from Trp and incorporation into thiostrepton.

Alternatively, quinaldic acid synthesis starts with *S*-adenosylmethionine-mediated methylation of Trp (Scheme 3) [52,66]. Deamination/oxidation steps follow and after ring opening, recyclization yields the quinaldic acid moiety. This is then reduced, attached to the structural peptide, and epoxidized. Upon epoxide opening, the second macrocycle of thiostrepton is formed.

**Scheme 4:**
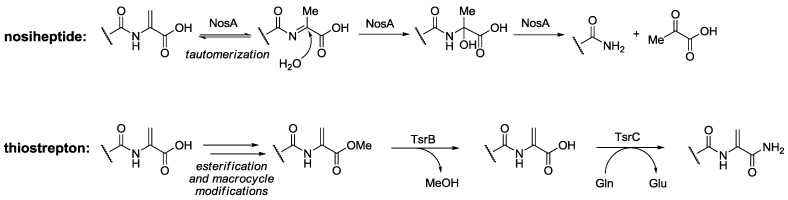
Proposed mechanisms for *C*-terminal amide formation during nosiheptide and thiostrepton maturation.

*C*-terminal tailoring is one of the last steps in thiopeptides maturation. In those cases where a *C*-terminal amide is present, two distinct mechanisms have been described for its formation (Scheme 4). Nosiheptide structural peptide contains an extra *C*-terminal Ser residue, which is lost during tail maturation, giving rise to a *C*-terminal amide [60]. By contrast, structural peptide of thiostrepton does not contain any extra amino acids and its C-terminal Ser residue can be methylated to form the corresponding ester (Scheme 4). The *C*-terminal amide is formed by deesterification and subsequent amidation using Gln as nitrogen donor [61].

### 2.3. Biological Activity

Thiopeptides are best regarded as antibacterial agents, however, their therapeutic potential is surprisingly broad and have been found to posses anticancer [76,77,78,79,80,81,82], antiplasmodial [83,84,85,86,87,88], immunosuppressive [89], renin inhibitory [90], RNA polymerase inhibitory [91], and antifungal [92] activities. This wide variety of biological functions has resulted in a very prolific literature outcome, positioning the macrocyclic scaffold of thiopeptides as a veritable privileged structure.

#### 2.3.1. Antibacterial Activity

**Figure 5:**
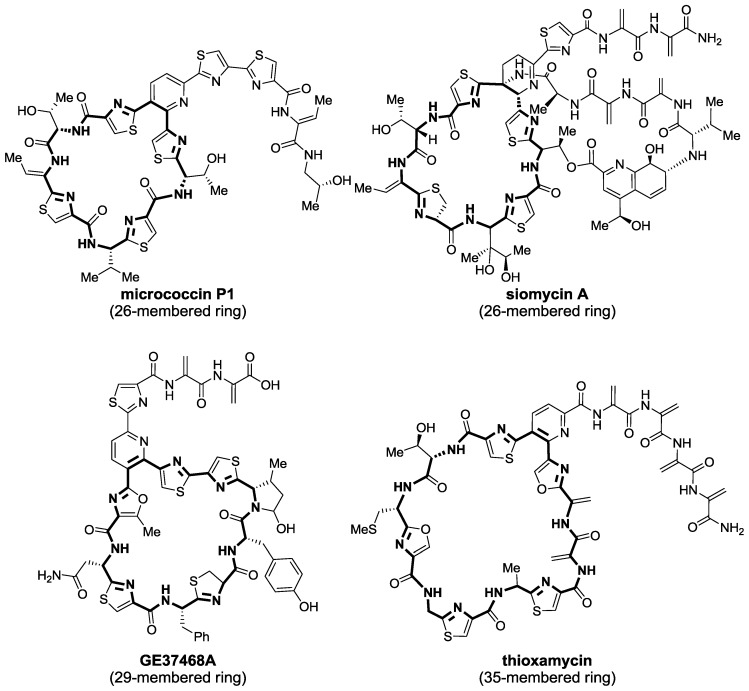
Thiopeptides have macrocycles of different sizes that determine their mode of action.

It is already well established that thiopeptides exert their antibacterial function via the inhibition of ribosomal protein synthesis. However, this is the result of different mechanisms of action that depend on macrocycle size. Thiopeptides exhibit macrocycles of three different sizes, 26-, 29-, and 35-membered rings, depending on the number of residues present. On one hand, thiopeptides of 26-member macrocycles, such as that of micrococcin P1 and the siomycins (Figure 5), are known to bind the GTPase-associated region of the ribosome/L11 protein complex. By doing so, the thiopeptide blocks the binding region of elongation factor G (EF-G) and does not allow translocation of the growing-peptide/tRNA complex in the ribosome to occur [93,94,95]. On the other hand, those thiopeptides with a 29-membered ring, in the fashion of GE37468A, bind to elongation factor Tu (EF-Tu), blocking its tRNA/amino acyl complex binding site [96,97,98]. As a consequence, the complex cannot be delivered into the ribosome and peptide elongation does not take place. Compounds with the largest macrocycles, those with 35-membered rings, maintain potent antibacterial activity; however, their molecular target still remains unknown.

**Figure 6:**
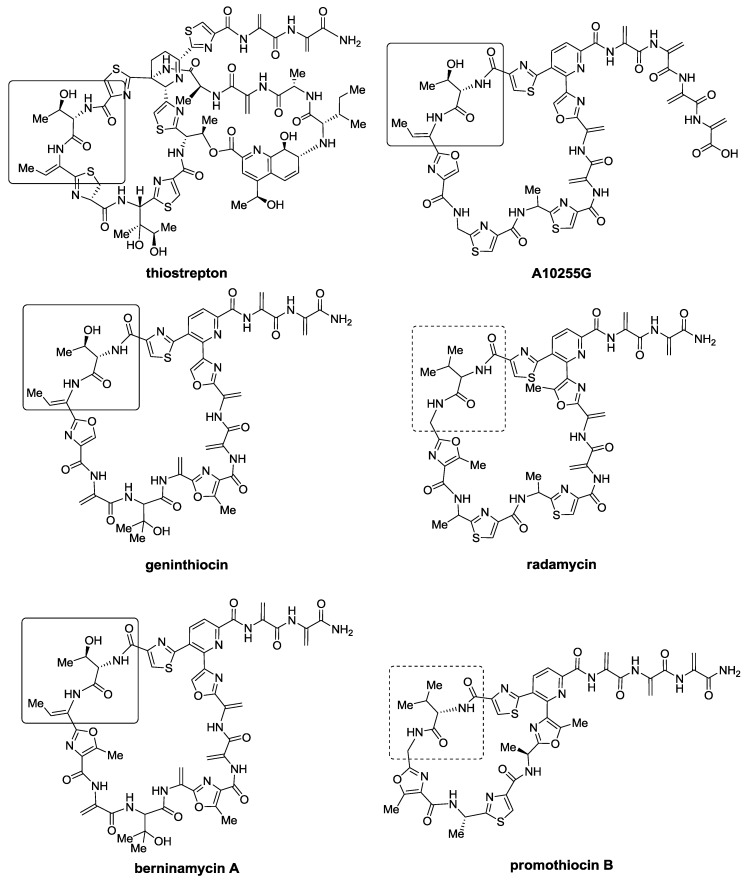
Thiopeptides with *tipA* promoting activity. A very preserved region, which has been shown to interact with the ribosome/L11 complex, is highlighted (solid squares). Radamycin, devoid of antibacterial activity, has a mutated sequence in the previously mentioned region (hashed squares). Promothiocin B possesses the same mutated residues, but maintains antibacterial activity, though in a 26-membered macrocycle.

Somewhat related to antibacterial activity is *tipA* gene promotion, which encodes two thiostrepton-induced proteins (Tip), TipAL and TipAS [99]. The latter, TipAS, serves as a mechanism of defense for bacteria, as it sequesters and covalently binds a thiopeptide molecule, which can no longer inhibit ribosomal protein synthesis. *TipA* promotion has been used to identify thiopeptides in a high-throughput screening program, which detected transcription of the promoter of *tipA* (*ptipA*) and led to the discovery of geninthiocin [100] (Figure 6). Other thiopeptides, such as thiotipin [46] and thioxamycin [42], and promothiocins [101] were also discovered thanks to their *tipA* promoting activity. Interestingly, the 35-membered thiopeptide radamycin is completely devoid of antibacterial activity, but is a very strong inducer of *tipA* gene expression (Figure 6). Various *tipA* promoting thiopeptides are depicted in Figure 6, where very preserved regions, associated with key interactions for binding with ribosome/L11 complex [102], are highlighted. Although those residues are different in radamycin, promothiocin B displays those same, not-preserved residues in a smaller 26-membered macrocycle and retains potent antibacterial activity. Apparently, *tipA* promotion activity is more dependent on the presence of a dehydroalanine-containing tail close to the six-membered central scaffold [103].

**Figure 7:**
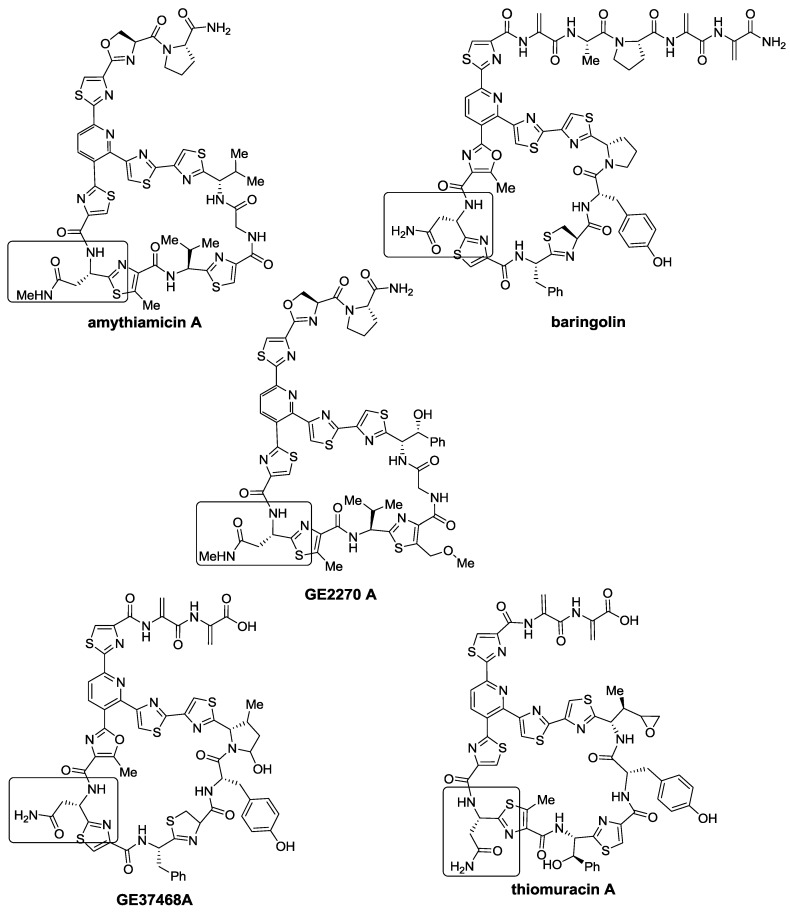
29-membered thiopeptides with a very preserved Asn residue highlighted with a square.

Thiopeptides known or presumed to bind to EF-Tu, those with 29-membered macrocycles, also possess a very preserved residue in their macrocycle, pointing to a key interaction required to exert their biological activity. The amythiamicins, baringolin, the GE2270 series, GE37468 A, and the thiomuracins, all belonging to the *d* series, preserve an Asn residue in the same position that is either methylated or not (Figure 7). Codon randomization experiments by Walsh *et al.* [104] produced various GE37468 A analogs that substituted Asn with Ala, Cys, His, and Ser; however, none of these analogs retained antibacterial activity, supporting the key role of this residue. Apart from providing a key contact with EF-Tu, the Asn residue participates in the stabilization of the bioactive conformation of the macrocycle, providing a transannular H-bond [105].

#### 2.3.2. Other Activities

One of the biological properties of thiopeptides of major interest, apart from the antibacterial one, is anticancer activity. In this regard, thiostrepton was found to selectively kill cancer cells without showing any cytotoxicity against healthy tissues [106]. Such a promising effect has been demonstrated to arise from selective inhibition of transcription factor forkhead box M1 (FOXM1) [78,81,82]. FOXM1 overexpression is associated with the development and progression of cancer and its selective targeting is a very large achievement, as transcription factors have been considered undruggable for a long time [107,108].

During synthetic efforts of Nicolaou’s group, it was discovered that the central core of thiostrepton (**1**) retained some antibacterial, but an increased potency against all cancer cell lines tested (Table 1) [76].

**Table 1:** Thiostrepton fragment **1** outperforms its parent compound against various cancer cell lines. 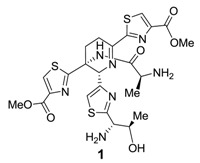

Compound	LC_50_ (μM)					IC_50_ (μM)			
	NCI-H460	HCT-116	SK-OV-3	MCF-7	K-562	1A9	PTX10	A8	AD10
thiostrepton	1.5	1.6	2.8	3.8	1.7	0.96	1.1	0.9	91.0
**1**	0.9	0.6	1.2	0.9	0.8	0.07	0.1	0.2	0.4

Many thiopeptides have been found to possess anti-malaria activity [83,84,85,86,87]. Although *Plasmodium falciparum* parasite cell is eukaryote, thiopeptides target apicoplast protein synthesis [109], which resembles that of prokaryotic organisms. Although it has been demonstrated that thiostrepton binds to the apicoplast 23S rRNA [83], thiopeptides of the *d* series, such as micrococcin P1 and amythiamicin A, are much more potent inhibitors of *P. falciparum* growth [84].

Very recently, the use of thiostrepton semi-synthetic analogs has demonstrated that it targets both the apicoplast ribosomes and the proteasome of *P. falciparum* [88]. This dual mode of action could make thiostrepton and similar thiopeptides less prone to resistance development than single-target drugs.

A screening program in search of immune-suppressants identified siomycin as inhibitor of antibody production by murine B-cells [89]. Comparison with thiostrepton showed the superior behavior of the structurally similar siomycin. Both thiopeptides are thought to possess a different mechanism of action than that of FK506, a common immunosuppressant drug, and would act directly on B-cells.

**Figure 8:**
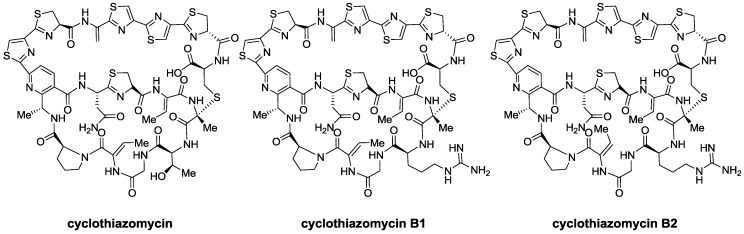
Cyclothiazomycin and its more recently isolated analogs B1 and B2.

Clyclothiazomycin is a very unique thiopeptide that does not possess a tail (Figure 8). In fact, the azole-containing branch that would serve as tail is linked to the macrocycle, forming a second ring, different from those found in thiopeptides of series *a*–*c* and *e*. However, due to its central tri-substituted pyridine ring, it is still considered a member of the *d* series. Perhaps due to its peculiar structure, different activities have been found for it. The first one to be described was human plasma renin inhibitory activity [90]. Renin is an enzyme associated with hypertension [110], diabetes [111], and Alzheimer’s disease [112] and is a rate-limiting enzyme in a cascade that starts with the cleavage of angiotensiogen and ends with the formation of angiotensin II. Due to this, renin is regarded as one of the most effective targets to treat hypertension.

Thiopeptides with a very similar structure to cyclothioazomycin were recently isolated and characterized and were named after the parent compound (Figure 8) [91]. In particular, cyclothiazomycin B1 was found to inhibit transcription by bacteriophage RNA polymerase. Such result might serve to further understand transcription at the molecular level.

Cyclothiazomycin B1 (Figure 8) also exhibits antifungal activity and inhibits the growth of various filamentous fungi. It presumably does so by binding to chitin, causing cell wall fragility [92].

Saramycetin has also been described as an antifungal thiopeptide [113]; however, it was not fully characterized and might not fulfill the structural requirements to fall into this family of natural products.

### 2.4. Conformation and Binding

The structure of thiopeptides has been studied using different techniques, which have also been used to elucidate the active conformation and the key contacts required to exert their biological activity. Though thiopeptides are relatively large molecules with big macrocycles, they possess many azoles, dehydroamino acids and amide bonds that confer the required rigidity for efficient binding [114]. First conformational studies were performed by NMR and provided solution structures of the promothiocins [115], nocathiacin I [47] and amythiamicin D [105]. For amythiamicin D, an intramolecular H-bond was detected [105], which seems to favor the bioactive conformation. The same H-bond interaction can be observed between the equivalent positions of active GE2270A and thiomuracin A analogs [116,117]. NMR studies of the thiostrepton/L11/rRNA complex [102] identified key contacts in the 26-membered ring of thiostrepton that led to the design of small analogs that maintained their binding capability [118].

X-ray analysis of the L11/rRNA complex and superimposition with optimized thiopeptide structures have determined that compounds targeting this complex bind to a cleft between the ribosome and L11 protein [94,95,119]. This region, the so-called GTP associated center (GAC), is also the binding site of elongation factor G (EF-G), which is responsible for translocation during ribosomal protein synthesis.

**Figure 9:**
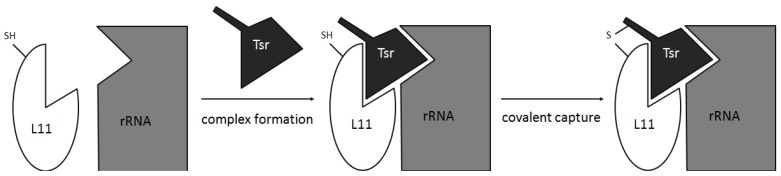
Proximity induced covalent capture. Mutated L11 protein has an external Cys residue in the area where interaction with the tail of thiostrepton is expected. Tsr = thiostrepton.

Alternatively, mutation studies have been carried out to study the thiostrepton/L11/rRNA ternary complex. Site-directed mutation of L11 to introduce a Cys residue in a suitable surface position, permitted proximity induced covalent capture (PICC) experiments to be carried out (Figure 9) [93]. In a PICC experiment, the newly introduced Cys should perform a 1,4-conjugated addition to a Dha residue of the thiopeptide tail. These experiments suggested a slightly different binding mode for thiostrepton, which would not sit right inside the cleft between L11 and the 23S rRNA, but closer to the ribosome surface. Further mutation studies of either L11 or 23S rRNA were in agreement with this alternative binding mode, demonstrating that mutations on L11 did not avoid thiostrepton binding, whereas mutations on the ribosome diminished affinity between the complex and the thiopeptide [120].

### 2.5. Chemical Synthesis

The complex architectures and challenging structures of thiopeptides have gathered the attention of many groups that have envisioned different strategies to accomplish their syntheses [49]. Pioneering work by the groups of Kelly [121,122,123,124], Shin [35], Moody [125,126], Nicolaou [127,128], Ciufolini [36,37], and Bach [127,129,130] developed different methodologies that led to the total synthesis of various thiopeptides. However, most efforts have been devoted to the construction of the central polyheterocyclic core [121,122,123,124,131,132,133,134,135,136,137,138], which have been synthesized by two well-distinguished main strategies: modification of an existing pyridine and construction of the central ring.

#### 2.5.1. Modification of Pyridine

**Scheme 5:**
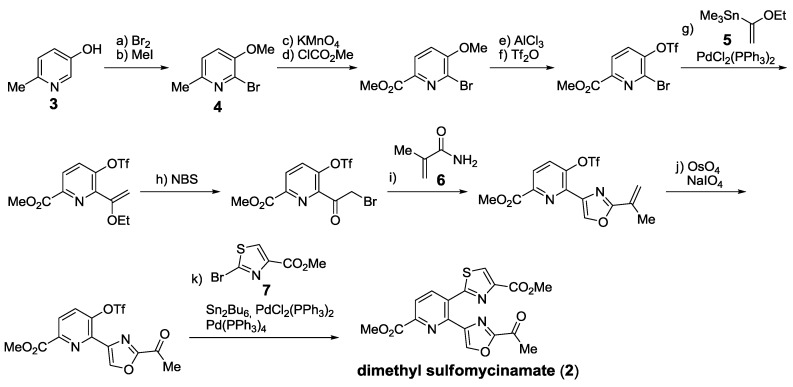
Kelly’s synthesis of dimethyl sulfomycinamate. Reagents and conditions: (**a**) Br_2_, pyridine, rt, 77%; (**b**) MeI, K_2_CO_3_, acetone, reflux, overnight 88%; (**c**) KMnO_4_, 90 °C, 3 h; (**d**) ClCO_2_Me, Et_3_N, DMAP, CH_2_Cl_2_, 0 °C to rt, 2 h, 65%; (**e**) AlCl_3_, CH_2_Cl_2_, reflux, 2 days, 93%; (**f**) Tf_2_O, 2,6-lutidine, CH_2_Cl_2_, 0 °C, 5 min, 95%; (**g**) **5**, PdCl_2_(PPh_3_)_2_, 1,4-dioxane, 100 °C, overnight 97%; (**h**) NBS, THF, H_2_O, rt, 10 min, 95%; (**i**) **6**, THF sealed tube, 100 °C, 3 days, 65%; (**j**) OsO_4_, NaIO_4_, 1,4-dioxane, H_2_O, rt 3 h, 85%; (**k**) **7**, Sn_2_Bu_6_, PdCl_2_(PPh_3_)_2_, Pd(PPh_3_)_4_, LiCl, 1,4-dioxane, 100 °C, overnight, 35%.

Early efforts by Kelly’s group focused on the synthesis of different thiopeptide cores [121,122,123,124] (Scheme 5). One of his landmark achievements was the synthesis of dimethyl sulfomycinamate (**2**), a product of acidic methanolysis of sulfomycin [139]. Selective bromination of **3** provided pyridine **4**, which contained three well-differentiated positions. Stille cross-coupling with **5**, oxazole formation using acrylamide **6** and an *in situ* stannation/cross-coupling protocol with bromothiazole **7** permitted the installment of the two azole rings present in **2**.

**Scheme 6:**
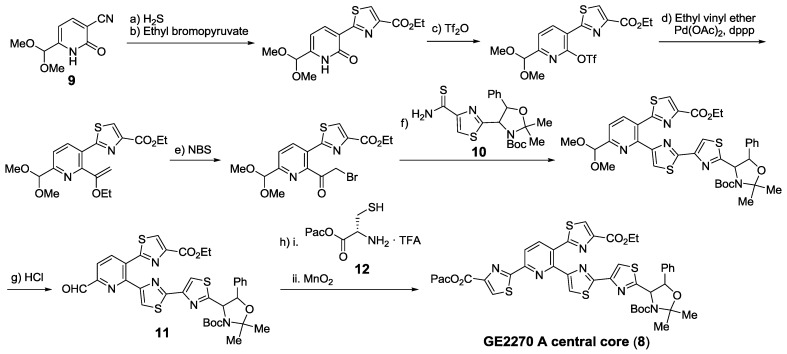
Shin’s synthesis of GE2270A central core. Reagents and conditions: (**a**) H_2_S, DMAP, Et_3_N, pyridine, rt, 3 days, 90%; (**b**) i. KHCO_3_, BrCH_2_COCO_2_Et, THF, 0 °C, then rt, overnight; ii. TFAA, pyridine, THF, 0 °C, 1 h, then rt, overnight, 53%; (**c**) Tf_2_O, DMAP, pyridine, 0 °C, 1 h, then rt, overnight, 93%; (**d**) ethyl vinyl ether, Et_3_N, dppp, Pd(OAc)_2_, toluene, reflux, overnight, 73%; (**e**) NBS, THF, H_2_O, rt, 5 min; (**f**) i. **10**, KHCO_3_, DME, 0 °C, 1 h, then rt, overnight; ii. TFAA, pyridine, 0 °C, 2 h, 63% (2 steps); (**g**) 2 M HCl in THF, rt, 24 h; (**h**) i. **12**, Et_3_N, toluene, rt, 15 min; ii. MnO_2_, toluene, rt, 12 h, 41% (2 steps). dppp = 1,3-bis(diphenylphosphino)propane; Pac = Phenacyl.

Shin and co-workers developed their own strategies and synthesized many polyheterocyclic cores and fragments, such as GE2270 A central core (**8**) [140,141,142,143,144,145,146,147]. Most of their syntheses start from a pre-functionalized pyridine or pyridone of the like of **9** (Scheme 6). For the synthesis of **8**, thiazoles were constructed using different methods, including Hantzsch syntheses with ethyl bromopyruvate and thioamide **10**, followed by condensation of aldehyde **11** with Cys **12** and subsequent oxidation with activated manganese (II) oxide.

**Scheme 7:**
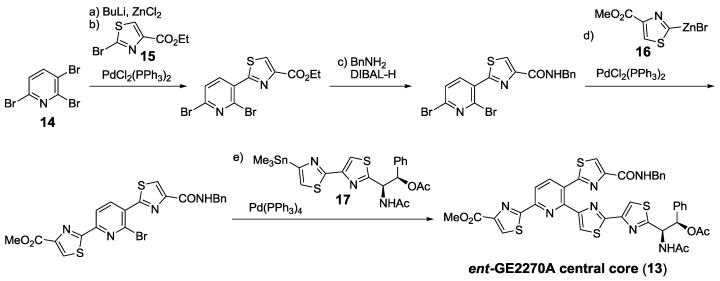
The synthesis of *ent*-GE2270A central core by Bach. Reagents and conditions: (**a**) BuLi, ZnCl_2_, THF; (**b**) **15**, PdCl_2_(PPh_3_)_2_, THF, 81% (2 steps); (**c**) BnNH_2_, DiBAL-H, THF, CH_2_Cl_2_, 86%; (**d**) **16**, PdCl_2_(PPh_3_)_2_, THF, DMA, 78%; (**e**) **17**, Pd(PPh_3_)_4_, 1,4-dioxane, 80 °C, 61%.

Bach’s group described a very convergent approach to the enantiomer of GE2270 A core (**13**), which started from 2,3,6-tribromopyridine (**14**), and was fully based on sequential cross-coupling reactions with thiazoles **15**−**17** [135] (Scheme 7). This work served for confirmation of the polyheterocyclic core stereochemistry and also set the methodology for further syntheses of GE2270A [129] and amythiamicins A and D [148].

**Scheme 8:**
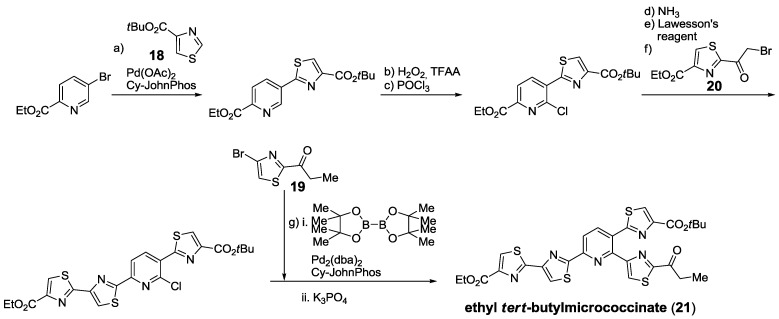
Synthesis of ethyl *tert*-butylmicrococcinate using direct C-H activation and one-pot borylation/cross-coupling of thiazoles. Reagents and conditions: (**a**) **18**, Pd(OAc)_2_, Cy-JohnPhos, Cs_2_CO_3_, DMF, 110 °C, 18 h, 74%; (**b**) CO(NH_2_)_2_, H_2_O_2_, TFAA, MeCN, 0 °C, 30 min; (**c**) POCl_3_, toluene, DMF, rt, 6 h, 78% (2 steps); (**d**) NH_4_OH, THF, rt, 82%; (**e**) Lawesson’s reagent, toluene, 2 h, 76%; (**f**) **20**, EtOH, THF (1:1), 65%; (**g**) i. **19**, bispinacolatodiboron, Pd_2_(dba)_2_, Cy-JohnPhos, KOAc, dioxane, 110 °C, 30 min; ii. K_3_PO_4_, dioxane, H_2_O, 110 °C, 12 h, 87%.

More recently, the group of Hoarau optimized conditions for the cross-coupling of thiazole-4-carboxylates (**18**) with pyridines using direct C-H activation on thiazole position 2 [137] and also formation of pinacolboronic acid and *in situ* cross-coupling on thiazole position 4, which is limited to 2-acylthiazoles, such as **19** [138]. Using, both, cross-coupling methodologies and Hantzsch thiazole synthesis with bromoketone **20**, the synthesis of ethyl *tert*-butylmicrococcinate (**21**) was achieved [138] (Scheme 8).

**Scheme 9:**
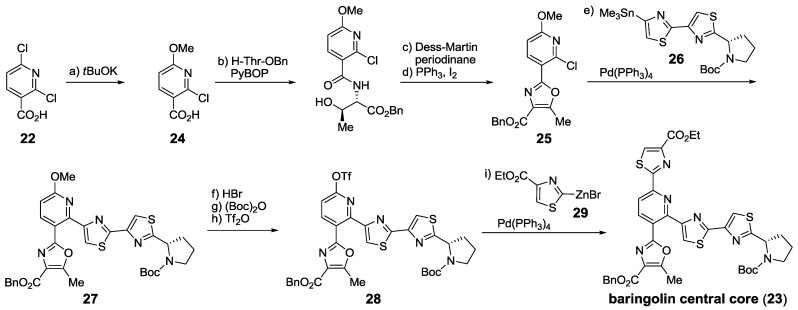
Synthesis of baringolin’s central polyheterocyclic core. Reagents and conditions: (**a**) *t*BuOK, MeOH, 65 °C, 4 days, 85%; (**b**) H-Thr-OBn, pyBOP, DIPEA, THF, 0 °C, 3 h, 89%; (**c**) Dess-Martin periodinane, CH_2_Cl_2_, rt, 6 h, 95%; (**d**) PPh_3_, I_2_, NEt_3_, CH_2_Cl_2_, 0 °C to rt, 15 h, 78%; (**e**) **26**, Pd(PPh_3_)_4_, 1,4-dioxane, 80 °C, 48 h, 88%; (**f**) HBr, AcOH, rt, 28 h, 73%; (**g**) (Boc)_2_O, NEt_3_, CH_2_Cl_2_, 0 °C, 4 h, 94%; (**h**) Tf_2_O, 2,6-lutidine, DMAP, CH_2_Cl_2_, 0 °C to rt, 3 h, 88%; (**i**) **29**, Pd(PPh_3_)_4_, DMA, 45 °C, 1 h, quant.

The last example of cross-coupling-based construction of thiopeptide polyheterocyclic cores was reported by Álvarez’s group [149] and used the regioselective differentiation of the two α positions in 2,6-dichloronicotinic acid (**22**) [150]. This strategy gave access to baringolin’s central core (**23**), facilitating its total synthesis [151] (Scheme 9). The carboxylic acid present in **24** was used to construct oxazole **25** and subsequent Stille cross-coupling with **26** [152] provided methoxypyridine **27**. After conversion of **27** to the corresponding triflate **28**, Negishi cross-coupling with **29** yielded **23**.

#### 2.5.2. Construction of the Central Ring

**Scheme 10:**
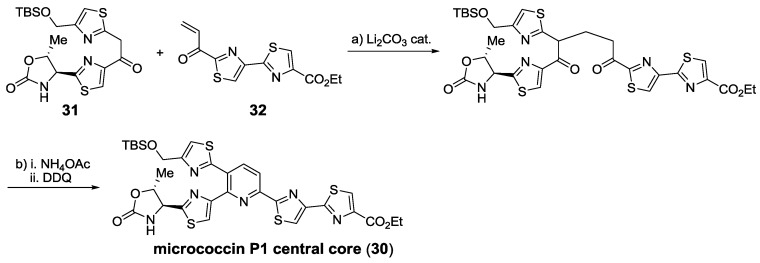
The synthesis of the polyheterocyclic core of micrococcin P1 by Ciufolini. Reagents and conditions: (**a**) cat. Li_2_CO_3_, EtOAc, 92%; (**b**) NH_4_OAc, EtOH then DDQ, toluene, 97%.

One of the most appealing contributions and the first one to rely on construction of the pyridine ring was Ciufolini’s synthesis of micrococcin’s central polyheterocylic core (**30**) [131] (Scheme 10). With the required thiazole building blocks, **31** and **32**, in hand, the pyridine ring is formed in two steps in almost quantitative yield. This led to the synthesis of the Bycroft-Gowland structure of micrococcin P1, which had been miss-assigned [36]. It took ten more years to finally synthesize the true structure of micrococcin P1 and confirm its stereochemistry [37]. Using the same strategy, thiocillin I has been recently synthesized [153].

**Scheme 11:**
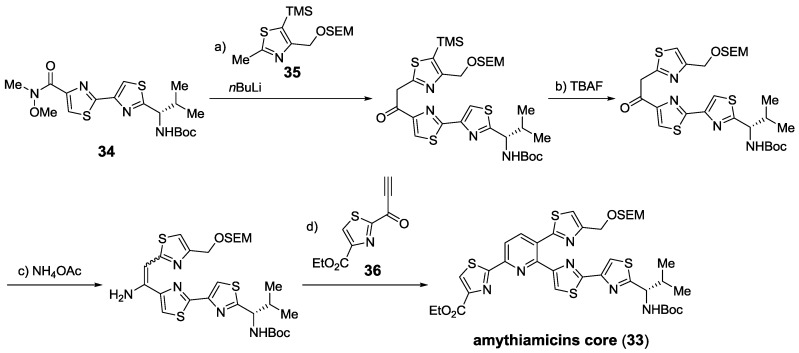
Synthesis of amythiamicins pyridine cluster using Bohlmann-Rahtz pyridine formation. Reagents and conditions: (**a**) **35**, *n*BuLi; H_2_O, 91%; (**b**) TBAF, THF, rt, 1 h, 93%; (**c**) NH_4_OAc, microwave, 120 °C (100 W), toluene, 30 min, 76%; (**d**) **36**, EtOH, 60 °C; toluene, AcOH, 70 °C, 85% (93% *ee*). SEM = 2-(trimethylsilyl)ethoxymethyl.

Bagley, first in Moody’s group, and co-workers also described their own syntheses of the thiopeptide cores, such as the amythiamicins core (33), constructing the pyridine ring with advanced thiazole-containing building blocks 34−36. Using Bohlmann-Rahtz pyridine synthesis, various thiopeptide cores were obtained [134,154,155,156,157,158,159] (Scheme 11). In addition, a very early total synthesis of promothiocin A was achieved [125,160,161].

**Scheme 12:**
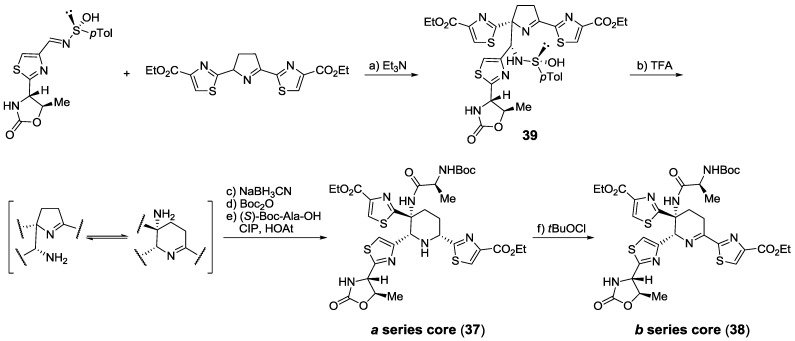
Synthesis of *a* and *b* series central core by Hashimoto and co-workers. Reagents and conditions: (**a**) Et_3_N, THF, −25 °C, 71%; (**b**) TFA, EtOH; (**c**) NaBH_3_CN, AcOH, EtOH, 52% (2 steps); (**d**) Boc_2_O, DMAP, Et_3_N, THF, 0 °C, 84%; (**e**) (*S*)-Boc-Ala-OH, CIP, HOAt, DIPEA, CH_2_Cl_2_, 93%; (**f**) *t*BuOCl, THF, −78 °C, then cat. DMAP, Et_3_N, 95%. CIP = 2-chloro-1,3-dimethylimidazolidium hexafluorophosphates.

The first synthesis of a polyheterocyclic core from the *a* (**37**) and *b* (**38**) series was reported by the group of Hashimoto. Their approach was based on formation of a substituted central 1-pyrroline **39** that underwent ring-expansion to form a piperidine ring **37** (*a* series) that could be selectively oxidized for form the corresponding 2,3,4,5-tetrahydropyridine **38** (*b* series) [162] (Scheme 12). In further reports they described the synthesis of other fragments [163,164] of *b* series thiopeptides and finally achieved the total synthesis of siomycin A [165,166].

**Scheme 13:**
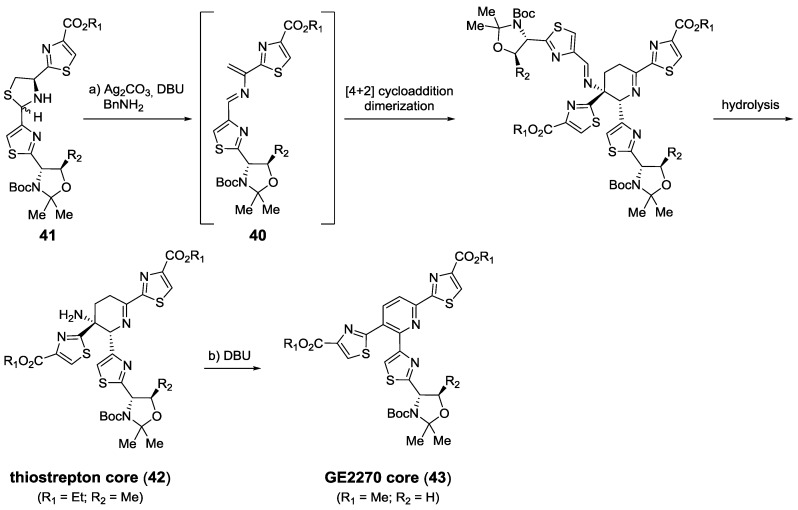
Bio-inspired synthesis of *b* and *d* series polyheterocyclic cores. Reagents and conditions: (**a**) Ag_2_CO_3_, BnNH_2_, DBU, pyridine, −15 °C, 1 h; then H_2_O/EtOAc (1:1), 1 h, 60%; (**b**) DBU, EtOAc, reflux, 5 h, 50%.

Cycloadditions have also been used for the synthesis of various central fragments. Almost simultaneously, the groups of Nicolaou [167] and Moody [132] developed two different bio-inspired strategies to obtain their target cores via an aza-Diels-Alder reaction. Nicolaou’s strategy was based on the silver-promoted formation of an aza-diene **40** from a precursor thiazolidine **41**, which would dimerize in a [4 + 2] cycloaddition (Scheme 13). The 2,3,4,5-tetrahydropyridine **42** (*b* series) thus obtained after hydrolysis of the cycloaddition adduct could be oxidized to form the corresponding fully unsaturated *d* series core (**43**).

**Scheme 14:**
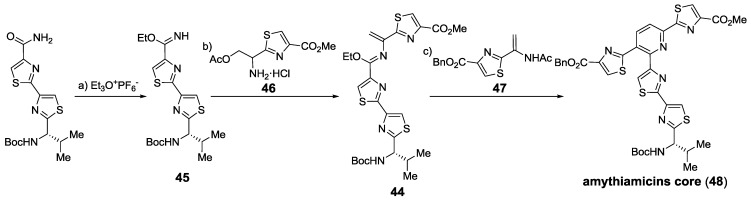
Bio-mimetic aza-Diels-Alder synthesis of 2,3,6-trisubstituted pyridine cores. Reagents and conditions: (**a**) Et_3_O^+^PF_6_^−^, CH_2_Cl_2_, 100%; (**b**) **46**, CH_2_Cl_2_, then DBU, CHCl_3_, 63%; (**c**) **47**, toluene, microwave, 120 °C, 33%.

Using Nicolaou’s methodology various total syntheses were achieved, including the challenging thiostrepton [127,128,168], but also GE2270A and GE2270T [169], and various amythiamicins [170]. On the other hand, the strategy by Moody relied on a previously formed aza-diene **44**, formed from the condensation of thiazoles **45** and **46** that could react with a different dienophile (**47**), yielding the aromatized cycloaddition product **48** [126,171] (Scheme 14).

**Scheme 15:**
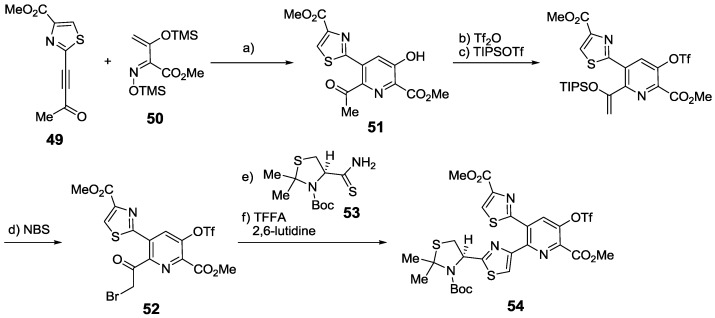
Synthesis of nosiheptide core via an aza-Diels-Alder cycloaddition. Reagents and conditions: (**a**) toluene, 180 °C, 55%; (**b**) Tf_2_O, Et_3_N, CH_2_Cl_2_, 0 °C; (**c**) TIPSOTf, Et_3_N, CH_2_Cl_2_,0 °C, 79% (2 steps); (**d**) NBS, THF/ H_2_O, 97%; (**e**) **53**, KHCO_3_, THF, −40 °C; (**f**) TFAA, 2,6-lutidine, −20 °C, 60% (2 steps).

Alternatively, Arndt designed another aza-Diels-Alder strategy that was not based on the biosynthetic pathway, but used an alkyne **49** and a protected α,β-unsaturated oxime **50** as starting materials, giving access to 3-hydroxypyridines, such as **51**. After a modified Hantzsch thiazole synthesis between **52** and **53**, a precursor of the central core of nosiheptide (**54**) could be accessed [136] (Scheme 15). Using this strategy, the main macrocycle of nosiheptide could be synthesized [172].

**Scheme 16:**
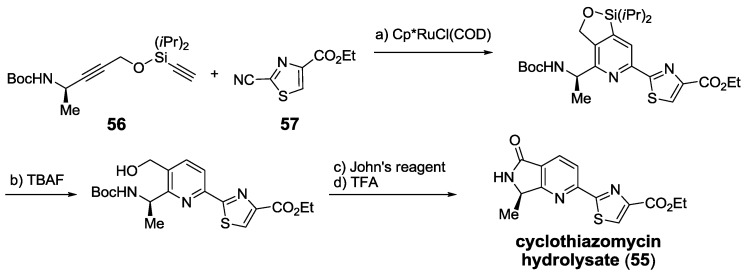
Syntehsis of cyclothiazomycin central core hydrolysate via a [2 + 2 + 2] ruthenium-catalyzed cycloaddition. Reagents and conditions: (**a**) Cp*RuCl(COD), 1,2-dichloroethane, 60 °C, 82%; (**b**) TBAF, THF, rt, 97%; (**c**) John’s reagent, acetone, 0 °C to rt; (**d**) TFA, CH_2_Cl_2_, rt, 80% (2 steps). Cp* = pentamethylcyclopentadienyl. TBAF = tetrabutylammonium fluoride.

Finally, one of the newest and most innovative contributions to the field is the synthesis of the central core of cyclothiazomycin hydrolysate **55** through a [2 + 2 + 2] ruthenium-catalyzed cyclotrimerization reaction between **56** and **57** [173] (Scheme 16). This yielded a product identical to that previously described by Bagley, thus confirming its identity [158].

Future advances in the preparation of substituted pyridines will surely facilitate the total synthesis of thiopeptides that still pose a challenge, but most importantly, will give easy access to fragments and analogs for the development of thiopeptide-based drugs with an improved pharmacokinetic profile.

### 2.6. In the Market

To date, only two thiopeptides have hit the market, thiostrepton and nosiheptide. Although both are exclusively devoted to veterinary use, their applications are very different. The lack of thiopeptide formulations for human use stems from their low aqueous solubility, a limitation that also restricts their use in animals.

Thiostrepton is used as one of the ingredients of an ointment for the treatment of cats and dogs skin infections. This ointment always has the same formulation (nystatin, 100,000 units; neomycin sulfate, 2.5 mg; thiostrepton, 2500 units; triamcinolone acetonide, 1 mg) and is sold by different companies with a variety of brand names: Animax (Dechra), Resortin (Hannah), Panolog (Novartis), Dermalone (Vedco), *etc.*

Alternatively, nosiheptide, is used as an animal growth promoter. This application was first described shortly after this thiopeptide was first isolated [174]. Nowadays, tons of pre-mixed animal food with the antibiotic are produced and commercialized.

Given the bad pharmacokinetic profile of thiopeptides, various analogs have been produced, mainly to improve their aqueous solubility. LFF571 (Figure 1), developed by Novartis, is a semi-synthetic analog of GE2270A currently under clinical trials for the treatment of *Clostridium difficile* intestinal infections in humans [9,175,176].

When considering the great therapeutic potential of thiopeptides and the efforts carried out by many research groups, it can be expected that their discoveries will lead to the development of analogs suitable for the treatment of more threatening systemic infections. The discovery of new thiopeptides might also hold the key to the future development of these natural products into suitable drugs. Moreover, the still vastly unexplored oceans are likely to become a fruitful source of the new thiopeptides to come.

## 3. Summary

A promising new family of antibacterial compounds, possessing other interesting bio-activities has been revised. Pioneering works from their isolation until the arrival to the marked though its biosynthesis, bio-activity and synthesis show that the thiopeptide are a wide range of investigation for scientists from different areas.
